# Congenital Hyperinsulinism International: A Community Focused on Improving the Lives of People Living With Congenital Hyperinsulinism

**DOI:** 10.3389/fendo.2022.886552

**Published:** 2022-04-28

**Authors:** Julie Raskin, Tai L. S. Pasquini, Sheila Bose, Dina Tallis, Jennifer Schmitt

**Affiliations:** Congenital Hyperinsulinism International, Glen Ridge, NJ, United States

**Keywords:** congenital hyperinsulinism, hypoglycemia, rare disease, burden of disease, challenges, patient organization

## Abstract

Congenital hyperinsulinism (HI) is a rare disease affecting newborns. HI causes severe hypoglycemia due to the overproduction of insulin. The signs and symptoms of hypoglycemia in HI babies is often not discovered until brain damage has already occurred. Prolonged hypoglycemia from HI can even lead to death. Disease management is often complex with a high burden on caregivers. Treatment options are extremely limited and often require long hospital stays to devise. Cascading from suboptimal treatments and diagnostic practices are a host of other problems and challenges that many with HI and their families experience including continued fear of hypoglycemia and feeding problems. The aim of this paper is (1) to describe the current challenges of living with HI including diagnosis and disease management told from the perspective of people who live with the condition (2), to provide family stories of life with HI, and (3) to share how a rare disease patient organization, Congenital Hyperinsulinism International (CHI) is working to improve the lives of HI patients and their families. CHI is a United States based nonprofit organization with a global focus. The paper communicates the programs the patient advocacy organization has put into place to support HI families through its virtual and in-person gatherings. The organization also helps individuals access diagnostics, medical experts, and treatments. CHI also raises awareness of HI to improve patient outcomes with information about HI and prolonged hypoglycemia in twenty-three languages. CHI drives innovation for new and better treatments by funding research pilot grants, conducting research through the HI Global Registry, and providing patient experience expertise to researchers developing new treatments. The organization is also the sponsor of the CHI Collaborative Research Network which brings medical and scientific experts together for the development of a patient-focused prioritized research agenda.

## Congenital Hyperinsulinism

Congenital hyperinsulinism (HI) is a disease that causes severe hypoglycemia due to the overproduction of insulin (1–7). While the mechanism of disease depends on the subtype, in all people with HI, the close regulation of blood glucose and insulin secretion is lost. This is dangerous for newborns because it can cause an inadequate supply of fuel which is needed for growth and development (1–7). When newborns experience this lack of energy, seizures or even coma can occur. If severe hypoglycemia is prolonged, brain damage may occur which can cause permanent seizure disorders, learning disabilities, movement, vision disorders, developmental disabilities, cerebral palsy, and even death (2–10).

The worst outcomes of the disease, brain damage and death, are preventable if the baby’s hypoglycemia is discovered before it is prolonged (4–6). For some, damage from hypoglycemia can occur in the first days of life. Many born with the disease are found to have hypoglycemia and diagnosed with HI in the first days, weeks, or months of life. Others are diagnosed later in the first year, and some may not be diagnosed until later in life.

There are many HI subtypes, characterized by genetic type (known or unknown), whether the condition is persistent or transient, diffuse or focal, or if the patient responds to diazoxide (1–10). Prevalence is reported to be from 1 in 2,500 births to 1 in 50,000 births, depending on where the individual with HI is born (3–6, 11–13).

Once someone is diagnosed, an individualized management plan is developed over weeks or months. Typically, the patient is hospitalized during this period. Treatment options range from simple to complex. A subset of patients are treated and well-managed on diazoxide, an oral medication discussed later in the paper because of its unwanted side effects. For those who can access genetic testing, specialized imaging, and an experienced surgeon, a cure is possible for focal disease; in this type of HI, abnormal pancreatic beta cells are limited to a focal lesion surrounded by normal beta cells. For the others with the condition, disease management is complicated, including one or more of the following: use of off-label medications *via* monthly or multiple daily injections or insulin pump, background dextrose through a feeding pump and gastrostomy tube, partial or subtotal pancreatectomy, frequent eating, and activity restrictions. No matter the type of HI, frequent daily blood glucose level checks with a home glucometer, and often with a continuous glucose monitor (CGM) are part of daily management (3–6, 14).

For many, medical management lessens over time; sometimes this occurs in a matter of months, when the condition is considered transient. For others, stabilization does not occur until later in childhood. Still others need medical management for decades or a lifetime (3–6, 8–10, 14).

In addition to the management of blood glucose to avoid hypoglycemia, people with HI often have other physical, developmental, and psychiatric issues. Feeding problems resulting from disease management and the condition itself, neurodiversity, and developmental delays resulting from prolonged hypoglycemia, and frequent co-morbidities like epilepsy, compound the issue of managing hypoglycemia on a regular basis (3–6).

Adding to the medically complex side of HI is the frequent and persistent fear parents of children with HI have that their child will experience hypoglycemia even when the child is being treated for the condition (**Table 1**). Since brain damage occurs in a large subset of those with HI, these fears have a basis in reality. Taken altogether, living with HI can be all consuming and HI families often feel the condition rules their lives (6).

**Table 1:** Challenges of Living with HI.

The challenges of living with HI depend on the following factors:
Age of diagnosis Access to a multi-disciplinary team with CHI expertise Time spent inpatient until an acceptable individualized disease management plan is established Neurologic differences from prolonged hypoglycemia Frequency, severity, and duration of hypoglycemic events Difficulty feeding and eating Frequency of feeds Reliance on nasal-gastric or gastrostomy tube GI issues including pain, frequent vomiting, and constipation, etc. Presence of a syndrome or other major co-morbidities Availability of medication, blood sugar supplies, and continuous glucose monitoring (CGM) Stage of life (newborn, infant, toddler, school age child, teenager, young adult, adult, senior) Level of anxiety or depression of caregivers Resilience Support for caregivers and availability of home-nursing, when necessary Availability of quality food Socioeconomic level of family Extent to which normal activities are curtailed to maintain euglycemia or perceived threat of hypoglycemia Need for home-nursing care Childcare, quality of education and availability of educational and school support

## Congenital Hyperinsulinism International

In 2005, with the mission to improve life for people with HI, an international group of parents of children with HI, who met through a parent email group, founded a patient advocacy organization, Congenital Hyperinsulinism International (CHI). CHI has focused its work on providing support to families, raising awareness to improve knowledge of HI among medical professionals and the general public, and contributing to research for a better understanding of the condition for more advanced diagnostics, new treatments, and cures.

The CHI Board of Directors (BOD) is the governing board for the United States (US) based nonprofit. Today, the BOD is comprised of six mothers of children born with HI. Each member has a skill or experience key to sustaining the nonprofit. The BOD has fiscal responsibility for CHI and sets its strategic direction. CHI also has a highly active advisory board that includes leading medical and scientific experts. CHI’s globally focused programs are led by the CHI staff, which currently includes five full-time professionals. CHI is also well supported by a large group of volunteers.

**Figure 1:**
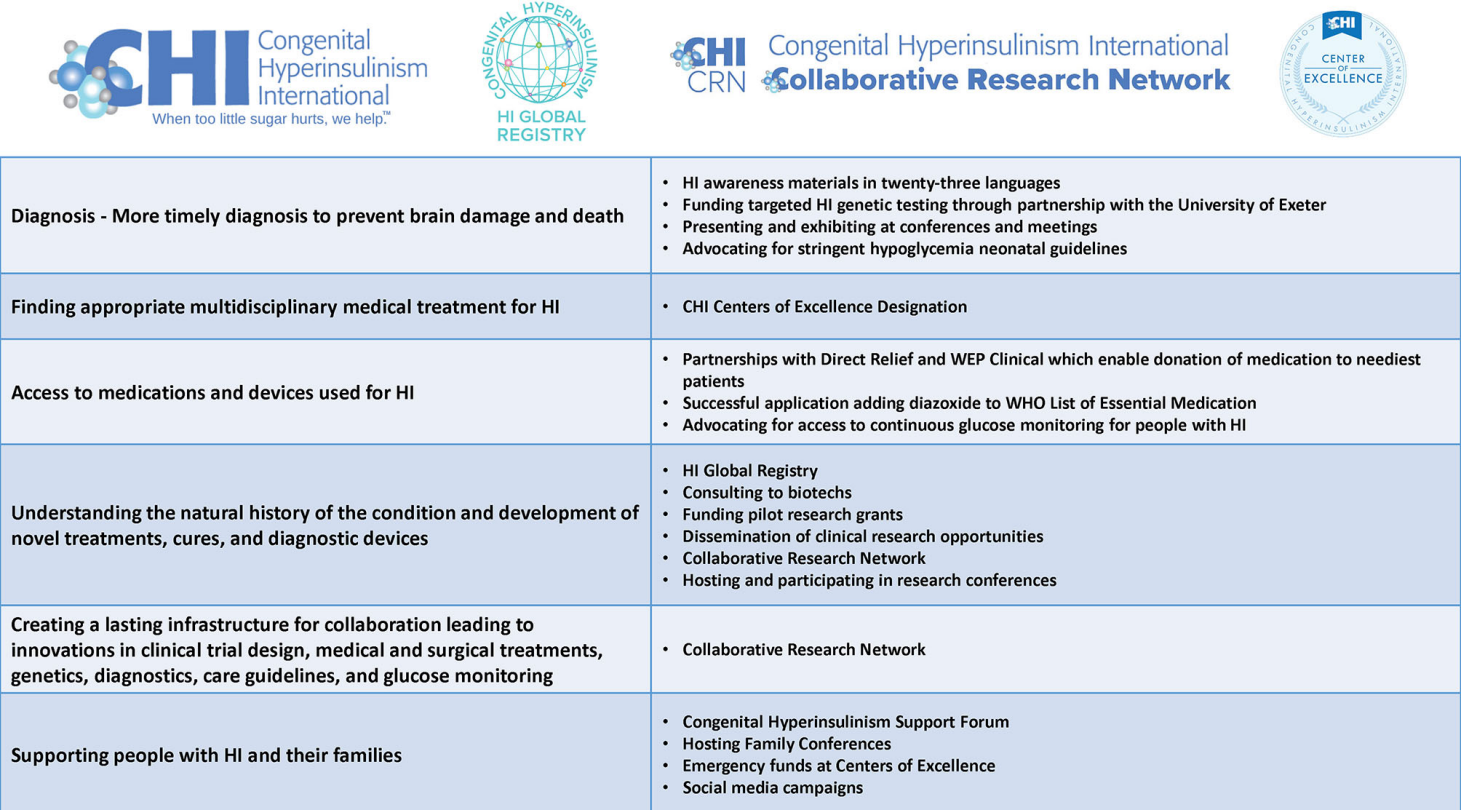
CHI Program Areas.

The aim of this paper is (1) to describe the current challenges of living with HI including diagnosis and disease management told from the perspective of people living with the condition, (2) to provide family stories of life with HI, and (3) to share how a rare disease patient organization, Congenital Hyperinsulinism International (CHI) is working to improve the lives of HI patients and their families. CHI is a United States based nonprofit organization with a global focus (**Figure 1**).

## HI Through a Patient Family Lens

To increase understanding of the experience of living with HI, to better understand the challenges, and as a personal counterpoint to the research and program work, which is described in the paper, three of the authors share their own family stories of diagnosis, finding treatment and care, and living and dying with HI.

### Parent of a Child With Hyperinsulinism-Hyperammonemia (HI/HA)

“There is nothing that can prepare one to travel the path of parenting a child with a rare disease. My daughter, now twenty-one, was officially diagnosed with HI/HA when she was fifteen months old. The journey to diagnosis was the scariest time of my life. Seizures, failure to thrive, inpatient stays, and monitoring at several children’s hospitals led to no answers. It was after these failed efforts that, as parents, we realized we had to search for answers on our own. We did and found answers across the country at the Children’s Hospital of Philadelphia (CHOP).

With a diagnosis and treatment plan identified, we resumed our daily lives with a slightly redefined focus. While no parent can ever identify, with certainty, the course of daily happenings of any child, the parents of a child with a rare disease face additional obstacles that include daily and lifelong challenges, personal and professional sacrifices, and feelings of guilt watching their child suffer.

With HI/HA, the most intense challenges revolve around unstable and unpredictable changes in blood glucose levels, dietary restrictions, availability of medication used to treat HI/HA, and a lack of knowledge or understanding of the long-term impact of HI/HA itself and the medication used for treatment.

In addition to producing excessive amounts of insulin inconsistently throughout the day, the HI/HA pancreas overproduces insulin when individuals eat protein, specifically the amino acid leucine. Finding the delicate balance of appropriate fasting times and balanced nutrition consumption can be unachievable. What works sometimes, does not work other times. Any internal or external shift in normalcy also impacts blood glucose levels. This includes teething, growth spurts, fevers, illness, puberty, excessive heat, excessive cold, exercise, etc. Therefore, the art of finding the balance constantly changes, making stable glucose levels nearly impossible.

Proglycem (diazoxide is the generic name) truly is what keeps our daughter alive and is the one approved medication that can help reduce insulin secretion. However, it is not effective for the hyperammonemia component of HI/HA. Therefore, individuals with HI/HA must be acutely aware of protein ingestion and there may be a neurologic consequence to elevated levels of ammonia. Protein is an integral nutritional necessity. When the individual with HI/HA is a child, there can be a high level of parental control over foods eaten; however, as an adult trying to integrate into general society, controlling protein intake as a component of HI/HA management has at times been traumatic for our daughter. It has created feelings of extreme self-consciousness.

Perhaps most worrisome, for a parent of a child with HI (HI/HA), is the unintentional lack of understanding of HI/HA for our children moving from childhood into adulthood. The HI medical professionals included in our child’s care are phenomenal. We owe our child’s life to them. But, when questions arise such as childbearing, long-term effects of being on high doses of diazoxide, and future medical issues seemingly unexplained, even the experts cannot provide insight or guidance. The unknowing initiates another layer of concern and worry for our 21-year-old female adult who is beginning to think of what life looks like in the future. Will she be able to have children (diazoxide has a warning that it cannot be taken when pregnant), will her children have this rare disease, how has diazoxide use for more than twenty years affected her body, are questions that remain unanswered.

The ongoing challenges combined with the more recent uncertainty about what the future will look like has led to noticeable changes in my daughter’s mental health and wellbeing. As parents, we prioritize continuing to seek answers, advocating for our daughter, and supporting all her medical, physical, and emotional needs that exist due to HI/HA.”

### Parent of a Child Born With Diffuse HI

“Despite my husband and I having prenatal genetic testing for the panel of diseases that are more common in Ashkenazi Jewish families, we did not discover we were carriers of disease-causing mutations in the ABCC8 gene until after our son was born. Our son was born 25 years ago and diagnosed in the second week of life.

His hypoglycemia symptoms were not recognized at birth or at any time while he was in the New York City birthing hospital despite my husband and I sharing our concerns about symptoms of extreme hunger, followed by lethargy and lack of interest in nursing. We were told by my obstetrician who came to the bedside in the newborn nursery, ‘he is a perfectly normal baby,’ and right on schedule, less than two days after giving birth, my son and I were discharged. Less than 24 hours after coming home from the birthing hospital, and less than 72 hours after being born, my son was readmitted to a New Jersey hospital, where he had numerous seizures upon arrival and his blood glucose level was so low it did not register. He was admitted to a NICU at this New Jersey hospital, and for a week the dedicated neonatologists performed many tests to find the source of persistent hypoglycemia (controlled with IV dextrose while testing to find the source). After several days, an experienced pediatric endocrinologist was consulted, and he made a clinical diagnosis of HI.

Once diagnosed, our son was transferred by ambulance to CHOP because of their expertise in treating children with HI. There he received superb treatment. After three months we finally brought him home. Throughout his life, he has continued to receive excellent care from CHOP. He continues his treatment there because there is no specialty care for adults born with HI in the US. His treatment for hypoglycemia due to HI included three subtotal pancreatectomies, background dextrose through a g-tube, and off-label use of octreotide and glucagon. He required private duty nursing at home for many years because he often needed urgent treatment for hypoglycemia day and night, even after surgeries and with medical treatment for HI (continuous glucose monitors did not yet exist). He had a brief period in his “tweens” when his blood glucose levels were in the normal range without medical treatment. At twelve years old, he became diabetic, which we knew would ultimately occur at some point because of his three subtotal pancreatectomies that removed 99% of his pancreas. The surgeries also caused permanent pancreatic insufficiency.

Prolonged hypoglycemia in the first three days of life caused my son to have irreversible disabilities. He has low vision from nystagmus and strabismus, fine motor-coordination issues, learning disabilities, and epilepsy. In the early years, life truly revolved around keeping his blood glucose levels in a safe range with medication and diet. Chronic constipation and stomach pain were often present. The combination of needing to eat to keep blood glucose levels in a safe range, to avoid additional neurological damage, combined with a lack of desire to eat, made meal and snack time fraught with anxiety for the whole family. We tried to normalize and accept the situation but there was always an underlying tension.

My son was able to attend excellent public schools in the town where we live. He was provided with accommodations, modifications, therapies, private nursing, and tutors that allowed him to learn alongside his typically developing peers. As a result of his excellent primary and secondary school education, he was able to go to college, one that supported his medical needs and learning differences. He graduated with a BA, with a concentration in education and psychology.

At 25 years old, He is trying to make the transition to independent living, which is more difficult due to his disabilities and health conditions secondary to HI. He currently lives at home and has a part-time job. He takes vigilant care of his health, is extremely focused on being physically fit, and leads a full and happy life, despite challenges. Being born with HI and having diabetes are intertwined into his identity, but they do not define him. He cares deeply about the acceptance of those who are neurodiverse and/or living with a rare disease. He has a regular practice of gratitude journaling. A caring extended family, close circle of friends, support from our local community, and close relationships with members of the CHI community have helped our son and our entire family to live a meaningful and happy life.”

### Parent of Children With Transient HI

“I am a parent of three children born with HI. The story of my children is rare and unique. Eleven years ago, shortly after being born, our first-born son was whisked away to the nursery because a nurse thought that he had low blood glucose. It turned out that she was right. After multiple rounds of testing on a neonate and 14-day stay in the NICU at a top hospital, we received a diagnosis of ‘transient hypoglycemia.’ We went home with this diagnosis, a glucometer, and a prescription for diazoxide. We were told our son needed to be fed regularly and that his little pancreas was overproducing insulin and causing his blood glucose to be low. Each day, before and after each meal, we checked our son’s blood sugar, poking his tiny heels with one hand and squeezing enough with the other to put a drop of blood on the glucometer, praying that it would read above 70mg/dL. This continued for about the first four months of his life. Our first son’s transient hypoglycemia was resolved by the time he was six months old.

Four years later, we welcomed our second son during a rare Nor’easter. They say that barometric pressure changes, induce labor, and I guess it was true; after 38 weeks and five days I went into labor on Valentine’s Day in the wee hours of the night. Upon arrival at the hospital, we stated that we needed to check the baby’s glucose when he was born; given the scenario we had with our first son, we wanted to make sure that our newborn did not have a similar issue with hypoglycemia. After many conversations with various staff including a pediatrician, we were assured that he ‘did not have what your older son had’ and we were released home. Going home with our newborn and this assurance made us feel ok. 36 hours later, we were back in the emergency room with a child that stopped breathing. We were told our newborn also had issues with his pancreas and an official diagnosis of HI. After learning about this diagnosis, we felt like our life was over. Was our firstborn really okay? Is this genetic? Why didn’t the doctors listen to us at the birthing hospital? How in the world did this happen to us? Shortly thereafter, our son died.

During my third pregnancy I was riddled with anxiety and didn’t have any comfort that I would get the appropriate care for her following birth. My daughter was born at one of the CHI Centers of Excellence at 39 weeks with a confirmed diagnosis of HI. She spent an additional week in the NICU and endocrine floor completing all the necessary testing. She too went home with a glucometer and had to be fed and checked almost every 3-4 hours. After her first 9 months, her transient hypoglycemia resolved, and she was “cured.” I am happy that she got a happy ending and now is a thriving 4-year-old child. This however, does not change our incalculable loss of our second son.

Following the events of our second son’s birth and subsequent death, I found the patient advocacy organization, CHI. Realizing our son’s death was 100% preventable made me want to do something to ensure that other parents did not experience such a loss. Timely diagnosis of HI and treatment could have saved my child’s life. Protocols that ensure neonates with a family history of low blood glucose are managed correctly need to be shared widely in the pediatric world. That is why I got involved with the advocacy organization CHI. The purpose of my involvement is to educate others by sharing our story and informing them that glucose is crucial to a newborn and that simple measures such as checking a blood glucose level could be life changing.”

These are just three examples of family experiences with HI. Each family history with HI is unique.

## Raising Awareness to Increase Timely Diagnosis

**Figure 2:**
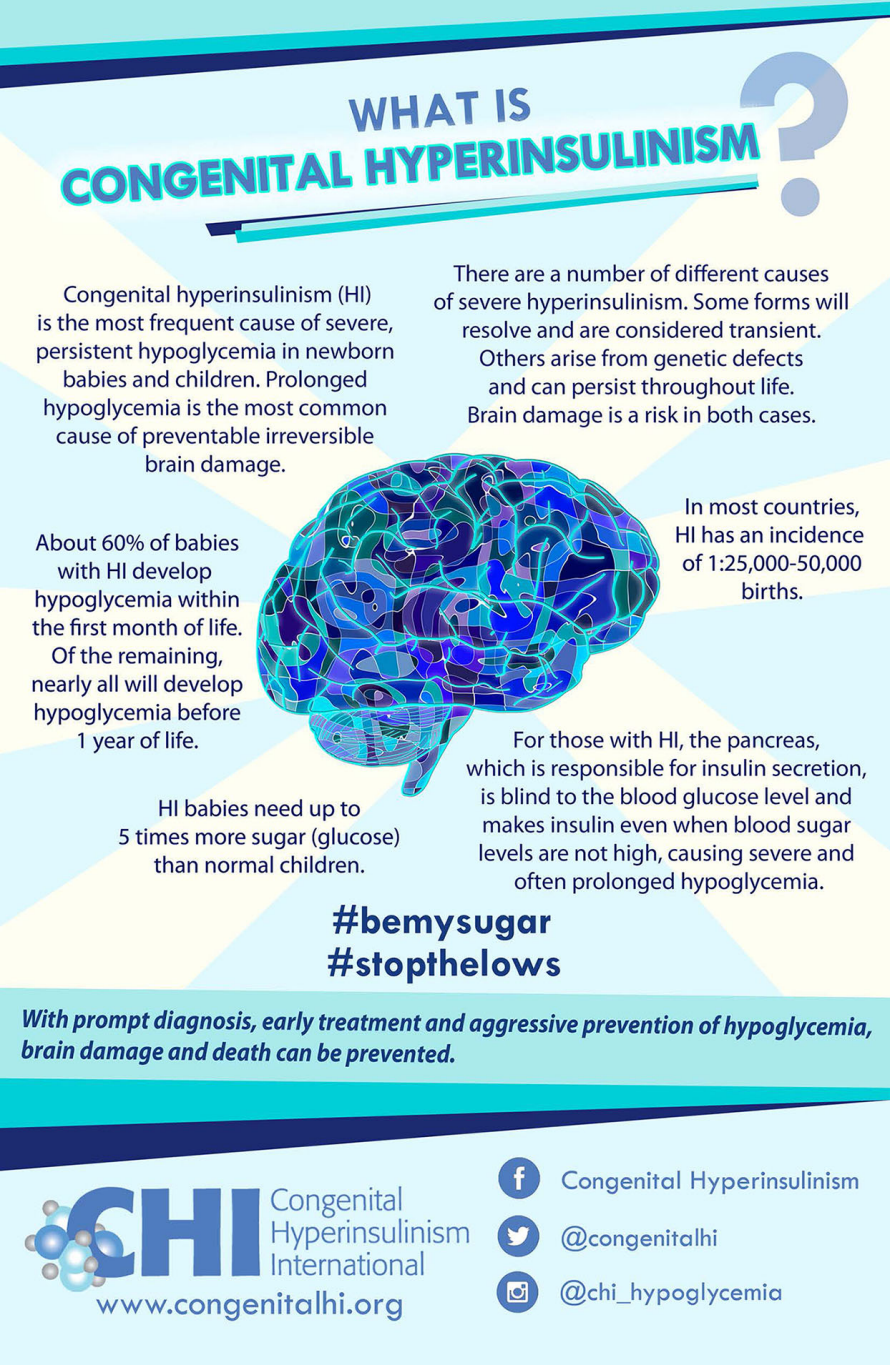
CHI Awareness Posters.

The family accounts in the previous section show the effect of delayed diagnosis and the harm it causes. With HI, “diagnosis in a timely manner” means uncovering hypoglycemia right after birth. CHI shares the hypoglycemia guidelines published by HI specialists in 2015 (15), and advocates for these guidelines to be adopted at individual institutions, to increase the number of neonates diagnosed and properly managed before brain damage or death occur. To increase knowledge of HI and hypoglycemia in the medical and general community, CHI has created the *What is HI?* and *The Signs and Symptoms of Hypoglycemia* awareness posters (**Figure 2**).

CHI posters are now available in 23 languages: Arabic, Bulgarian, Catalan, Chinese Simplified, Chinese Traditional, Czech, Dutch, English, French, German, Georgian, Greek, Hebrew, Hungarian, Italian, Polish, Portuguese, Russian, Serbian, Slovak, Spanish, Swedish and Turkish. CHI has also created smaller 4x6 inch card sized *Say Hi to HI* infographics that can be printed and shared easily with teachers, friends and family that explain HI, hypoglycemia, and its effects in the simplest terms. The educational postcards are available in English, French, German, Russian and Spanish. With these posters and postcards available in many languages, CHI is working to ensure that knowledge of the risks associated with prolonged hypoglycemia in newborns is available globally.

## Genetic Testing for Personalized Care

**Figure 3:**
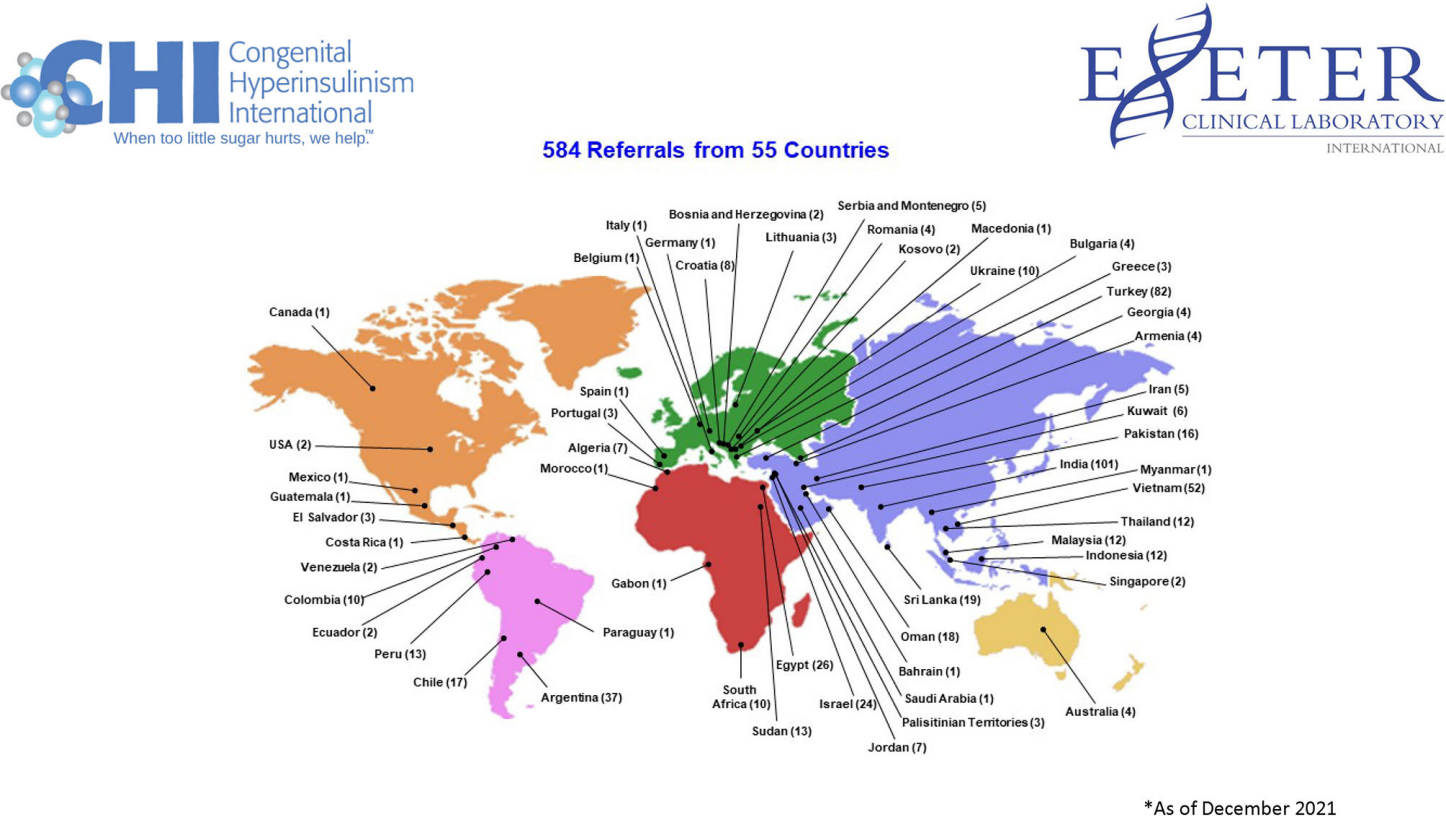
Exeter and CHI Referral Countries.

Genetic testing is often a crucial element in determining what is the best treatment for each person with HI. CHI funds targeted HI genetic testing done at the University of Exeter Clinical Laboratory in the UK. This laboratory is renowned for its groundbreaking work in HI genetics. With this program, genetic testing is free of charge to families from anywhere in the world who do not have this testing covered by a healthcare plan (**Figure 3**).

Between July 2018, when the program began, and December 2021 CHI has funded genetic testing for 584 individuals from 55 different countries across five continents with medically diagnosed HI (16). An additional 460 samples were received from family members. An example of the importance of genetic testing to the diagnostic process is that it offers valuable information about the likelihood of focal disease, which can be cured. Since the program’s inception, 69 patients have been found to have a single paternally inherited mutation in the ABCC8 or KCNJ11 gene which is evidence the condition may be focal in those children (16).

To further share knowledge of HI and neonatal and childhood hypoglycemia and to spread knowledge of the importance of timely diagnosis, CHI also exhibits at international medical conferences and engages in social media campaigns on four social media channels.

## The Search for Excellent Multidisciplinary Medical Care for HI

Once diagnosed, it is often a challenge to find medical professionals with enough knowledge of HI and a multi-disciplinary team to provide optimal treatment for it. To ensure HI families have information about where to go for comprehensive specialized HI medical care, CHI recognizes and designates expert centers, CHI Centers of Excellence (COE), that provide the highest level of multi-disciplinary care to HI patients and their families.

**Figure 4:**
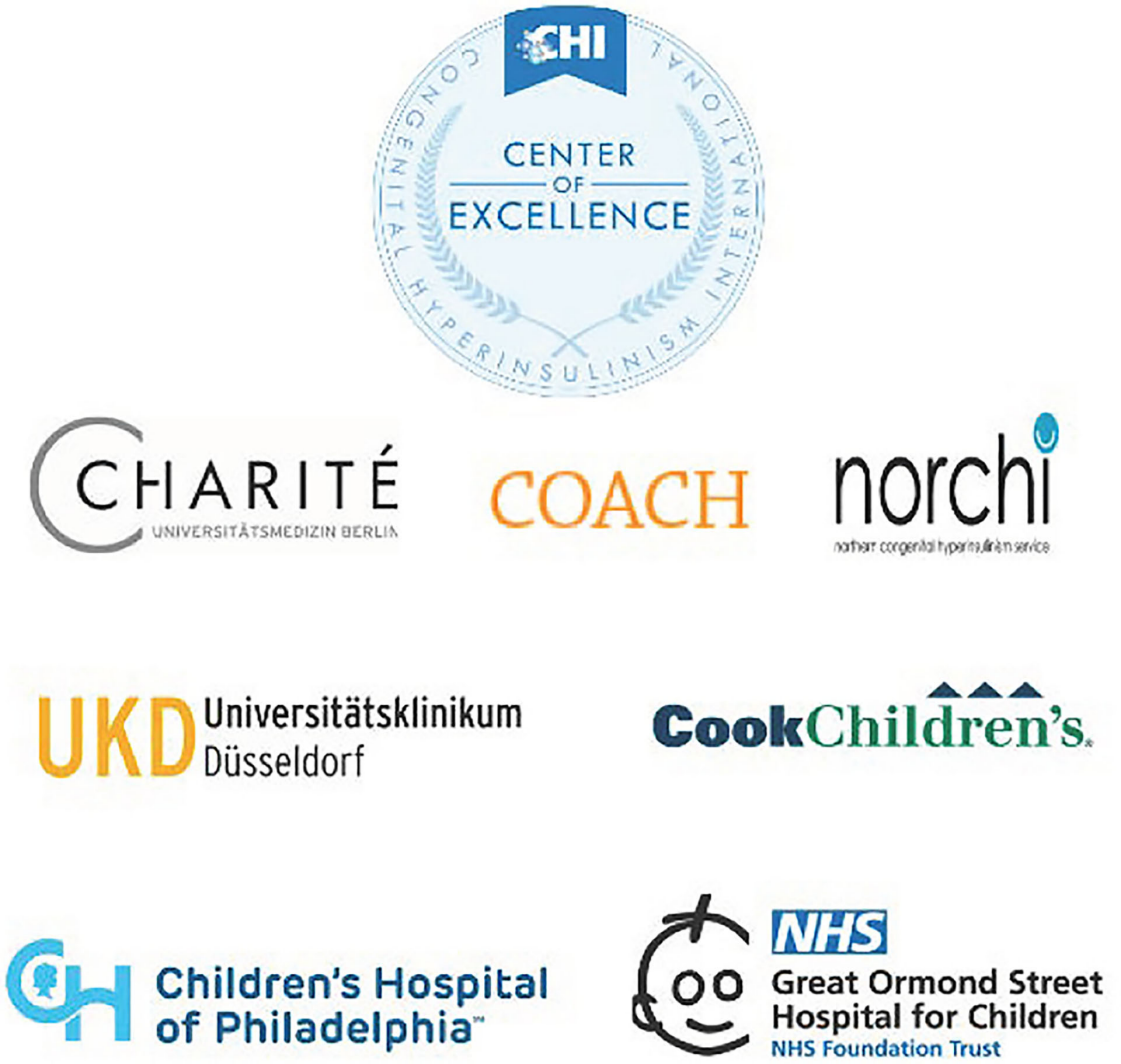
CHI Inaugural Centers of Excellence.

The CHI COE designation also recognizes an on-going commitment to research and collaboration (17). The first group of six centers was designated in 2021 (**Figure 4**). To receive the designation, centers completed an online application consisting of 33 elements including multidisciplinary expertise in fields such as surgery, gastroenterology, diet/nutrition and feeding, psychiatry, neurology, pathology, radiology, genetics, and neonatology. In addition to medical fields associated with being able to provide successful pancreatectomies, the other areas of expertise are necessary because there are often other health issues, in addition to hypoglycemia, that HI patients need addressed (17).

Centers that have received the designation are leaders in HI research, and provide continuing medical education at the local, national, and international level. For CHI has set up special support funds to help HI families with financial needs pay for transportation, food, and hotel expenses. Presently, funds for this purpose are available to families at CHOP and Cook Children’s Medical Center.

CHI also helps to facilitate international collaborations leading to standardized guidelines that support an individualized approach to care that will be supported by clinical outcomes evidence. CHI shares the patient family perspective for such projects and provides a virtual meeting place and infrastructure for collaboration.

## Supporting People With HI and Their Families

In addition to having an excellent care team, families affected by HI need ongoing support. Launched in 2011, the CHI Family Support Forum with over 1,900 members from 79 countries is a private online space where individuals affected by HI help one another cope with the stress of living with the condition and can share and receive feedback on the challenges or triumphs they experience at any given time or any topic about which they are searching for information (18).

The Forum is open to any parent of a child with HI and teenagers and adults with HI. Close family members can also join if the parents of the child with HI consent. Frequent topics include centers of excellence, feeding difficulties, child development, sharing feelings of depression or anxiety, genetics, side effects of medications, when and how to go back to work, symptoms comparisons, future pregnancies, and sharing milestones. Members understand that the forum’s purpose is to support and share experiences, and not to provide unsubstantiated medical advice. Members often post photos and videos. The Forum is searchable by topic.

In addition to the private CHI Family Support Forum on Facebook, CHI maintains meaningful connections to the HI community through the social media channels Facebook, Instagram, Twitter, and LinkedIn. On these channels, CHI shares research and advocacy news, clinical research opportunities, and HI patient and family stories. Through CHI social media channels, CHI regularly reaches newly diagnosed families. CHI also maintains a highly searched webpage on HI with 17,423 visitors from 155 countries in 2021 (19).

CHI family conferences are another way families can gain support, learn, and share their experiences. CHI has organized twenty-three international conferences in the US, Europe, and virtually. Frequent agenda topics are coping with the stress of living with a rare, chronic condition, diagnostic and cure fasts, “Ask the Experts,” understanding genetics, and learning about new investigational treatments. Increasingly medical professionals are joining the family conferences as participants, to learn about HI from the medical professional and scientist speakers, as well as to gain insight into the HI patient and family perspective.

## Rare Disease Medications and Devices Can Be Hard to Access

For many who respond to diazoxide or a form of octreotide, there are still significant hurdles to accessing these drugs. In 2017 and 2018, CHI surveyed 74 pediatric endocrinologists from 42 countries about access issues. The survey revealed that 64% of responding pediatric endocrinologist have patients who have trouble accessing diazoxide (20).

Later in 2018, with the hope of expanding diazoxide access, CHI supported an application for diazoxide to be added to the WHO list of essential medications. The application was approved and diazoxide is now on this list (21).

CHI has also developed relationships with the manufacturers of diazoxide and other drugs used off-label but regularly prescribed by HI doctors. Through collaborations with the global nonprofit Direct Relief and WEP Clinical. CHI has been able to facilitate regular donations to the neediest patients in areas where it is most difficult to obtain the medications. There are still many HI families who have trouble accessing diazoxide, which is why CHI works to increase market access and to expand donation programs.

Another access issue for people is that home devices for measuring blood glucose levels (home glucometers and CGMs) are not made or approved for people with HI. These devices are approved by regulatory bodies for people with diabetes.

CGMs have made living with diabetes far easier, and they have also improved disease management. A CGM gives the wearer continuous information about blood glucose levels rather than just one reading in time. In this way, CGM guides treatment and helps people with diabetes have better blood glucose control. Not having to prick their finger multiple times a day to measure blood glucose is also a major improvement in the lives of people with diabetes who choose and can access CGMs.

A subset of people with CHI have been able to obtain CGM devices and supplies including 44% (n=61) of participants in the HI Global Registry (14). According to experiences shared in the patient Forum those that do typically find them useful and reasonably accurate. A smaller group of HI families have tried CGMs and chosen not to use them routinely because they do not find they track close to home glucometer values, and in some cases the wearer experiences frequent compression lows (22).

CHI advocates for individuals with HI seeking to gain access to CGMs and glucometers and supplies when there are issues with healthcare plan coverage. CHI is also encouraging CGM research that will lead to regulatory approval of CGMs for people with HI and other rare hypoglycemia disorders. Universal access to devices that are convenient and accurately measure blood glucose levels are as necessary for HI as they are for diabetes.

## The Problems With Current Treatments and Diagnosis Uncovered in CHI’s HI Global Registry

**Figure 5:**
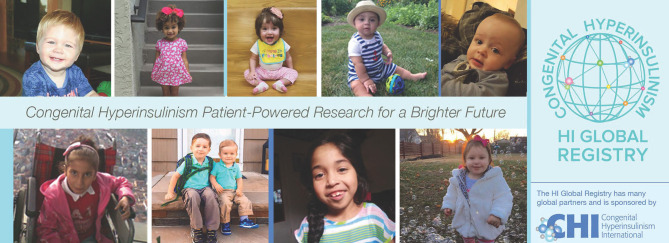
HI Global Registry Banner Image.

To accelerate the development of new treatments and cures, to increase timely diagnosis, and to improve care, and the understanding of HI, in 2018, CHI launched the HI Global Registry, a patient-reported IRB approved natural history study (**Figure 5**) (14). HIGR is hosted on the IAMRARE™ Platform which was developed by the National Organization for Rare Disorders with input from patients, caregivers, and government stakeholders to ensure a safe and user-friendly system for study participation (23). Members of a steering committee made up of leading international HI specialists and patient advocates advised on the development of the HIGR surveys and participate in future developments to strengthen it as a resource. The registry consists of a series of online surveys that ask the participant questions about the patient’s experience with the disorder over his or her lifetime. This information is then aggregated to produce research reports that can be studied by researchers.

As of September 1, 2021, HIGR has participants from 46 countries and a total of 335 enrollees. Participants can complete one or more of the thirteen surveys relating to the experience of living with HI. The patient reported data shared below reinforces clinician and academic research and provides new insights patients are uniquely positioned to share.

Forty-one percent (n=29) of 70 participants with diffuse HI report hypoglycemia defined as below 70 mg/dL or 3.9 mmol several times a week or more. Participants in this group include those taking diazoxide, octreotide, and lanreotide.

HIGR also collects data related to medication side-effects including an increase in body hair in 85% (n=93) of 110 participants taking diazoxide. This physical alteration can have a profound psychosocial impact on individuals, especially children.

In addition to on-going hypoglycemia and medication side-effects many HIGR respondents report other diagnoses or symptoms that impact their daily life. Of 124 respondents, 38% (n=47) report participants have a chronic neurologic problem which they feel is due to the participant’s prolonged hypoglycemia and 44% (n=57) report delays in meeting developmental milestones. Of 132 respondents, feeding issues were experienced by 69% (n=91) of participants with 40% (n=53) experiencing issues with appetite, and 39% (n=52) refusing to eat. The lack of desire to eat and the risks of not eating for those with HI produces a cycle of anxiety for both the HI child and caregivers. Thirteen percent (n=18) of 135 individuals who responded to the Other Diagnoses Survey report the participant has epilepsy. The frequency with which respondents report neurologic developmental issues reinforces the need for discovering and treating hypoglycemia before brain damage occurs.

The physical and psychological health of parents of children with HI are impacted by the stress of trying to maintain normal blood glucose and treatments that interfere with daily life. Parents of 48% (n=59) of the 123 participants report their physical health has suffered from having a child with a HI-related condition. Parents of 67% (n=83) of the 123 participants report their mental health has suffered from having a child with a HI-related condition. Individuals are included in this statistic if they answered that their health suffered “somewhat,” “quite a lot,” or “very much.”

Individuals who undergo a pancreatectomy are often trading one disease for another. All 9 people who report having diabetes had a pancreatectomy and five take pancreatic enzymes for pancreatic insufficiency. The other 12 individuals who underwent a subtotal pancreatectomy and do not report developing diabetes are under the age of 13. This shows the need for a treatment that would reverse diabetes and pancreatic insufficiency for those who have undergone subtotal pancreatectomy for HI.

Since HIGR was launched, 13 participants with a mutation in the GLUD 1 gene that causes HI/HA have been reported. People with HI/HA have hypoglycemia due to glutamate dehydrogenase (GDH) over-activity in the pancreatic beta cells, which can be treated with diazoxide. However, these patients also have other medical problems that may be due to GDH over-activity in other cell types causing seizures and developmental delays. These patients need a treatment to address the neurologic issues that are not caused by prolonged hypoglycemia.

As a rare disease patient organization, it is critical that CHI helps drive a patient-centric research strategy. Recruitment for the registry is on-going as additional patient participation can inform avenues for future research studies, identify areas of patient needs, and improve clinical care. It is especially important to detail the natural history for sub-group analysis of HI patients. Therefore, we encourage all individuals living with HI or their caregivers to join the registry and for clinicians to encourage their patients to participate.

## The Promise of Investigational Treatments

Beginning in 2015 there has been a significant increase in HI research in pre-clinical and clinical phases with the potential to lead to new treatments (24). These important research projects are taking place at academic institutions and commercial biotechnology companies. CHI supports four of these research studies by providing data from HIGR to help the researchers develop innovative and patient-informed clinical trial design.

CHI also supports clinical development programs by sharing the patient journey through presentations, discussions, and listening sessions. CHI also reviews protocols and informed consent forms. In addition, CHI informs the patient community of the importance of clinical research for the development of better diagnostics, new treatments, and cures

To further support the development of new treatments, better diagnostic tools and practices, and an improved quality of life, CHI has supported eight pilot research grants, seven through the Million Dollar Bike Ride (**Table 2**). The Million Dollar Bike Ride is a project of the Orphan Disease Center (ODC) at the University of Pennsylvania. With an annual bike ride in Philadelphia as a fundraising vehicle, dozens of rare disease teams raise funds for rare disease research. Each team determines a disease-focused research topic and submits language to the ODC for the Request for Applications. Each team provides a roster of potential reviewers with expert knowledge of the disease area. The ODC oversees the selection process, administers the grants, and matches a sizable portion of the funds raised (25). CHI has also administered one grant directly, the hyperinsulinism hyperammonemia pilot grant (26).

**Table 2:** Million Dollar Bike Ride and CHI International Pilot Grants.

Million Dollar Bike Ride Grants	
Year	Description
2014	Diva De Leon Crutchlow, MD, of the Children’s Hospital of Pennsylvania, was awarded $60,000 for a pilot study investigating the efficacy and safety of sirolimus in the treatment of congenital hyperinsulinism. This research was very important at the time because sirolimus was being prescribed off-label for patients with congenital hyperinsulinism, and there was a lack of research in this population.
2015	Mark Dunne, PhD, of the University of Manchester, received $71,000 for the topic: Toward Precision Medicine in the Treatment of Congenital Hyperinsulinism in Infancy. The focus was on expanding islet cell study to include not only the study of beta cells. This research is very important because current medications are often poorly tolerated, ineffective, with adverse effects.
2016	Changhong Li, MD, PhD, then at the Children’s Hospital of Philadelphia, was awarded $82,000 for Drug Development for treatment of Glutamate Dehydrogenase Hyperinsulinism. The goal of this study was to identify a safe medicine that will treat all the health issues resulting from activating mutations in GDH in people with GDH-HI. Dr. Li continues to research new treatments for GDH-HI as the Associate Director at Nanjing Institute of Advanced Biotechnology.
2017	Diva De Leon Crutchlow, MD, of the Children’s Hospital of Pennsylvania, received $87,109 to research the Bihormonal Bionic Pancreas for the treatment of Diabetes Post-Pancreatectomy in Children with Congenital Hyperinsulinism. She collaborated with Dr. Steven Russell of MGH and Dr. Ed Damiano of Boston University and Beta Bionics to examine the safety and efficacy of the Bionic Pancreas system in children and young adults with hyperinsulinism who have developed diabetes after pancreatectomy.
2018	Amanda Ackermann, MD, PhD, of the Children’s Hospital of Philadelphia, was the grantee. She received $84,080 for research on Vitamin E Supplementation in Hyperinsulinism/Hyperammonemia Syndrome. Vitamin E has been tested in human cell lines and mice with activating GDH mutations and shows potential promise as a treatment for those with GDH HI (HI/HA). The study looks to see if vitamin E supplementation is well tolerated and reduces hypoglycemia, hyperammonemia, and seizures.
2019	Thomas Smith, PhD, of the University of Texas, Medical Branch, received a grant of $72,014. Once again, the focus of this grant was “Towards new therapeutics treatment for hyperinsulinism/hyperammonemia syndrome (HI/HA). The focus here is on targeting GDH directly to treat all symptoms associated with HI/HA throughout the body.
2020	Indi Banerjee, MD, of the University of Manchester in the UK, received $73,190 for the research topic “Maximizing the utilization of the Hyperinsulinism Global Registry (HIGR).” His proposed study which includes partners from a number of institutions in Europe, the US, and Asia aims to “build on the opportunity to add medical grade information to existing parent reported HIGR information, thereby joining up clinical and parent perspectives in the search towards better understanding and improved treatment for HI. MaxHIGR will lay the basis for HIGR to evolve into a registry that will tell us about the natural history of disease, which treatments are better and have less side effects and how we can improve the quality of life of children and families living with HI.”
2021	Elizabeth Rosenfeld, MD, of Children’s Hospital, received $73,045 for a study on the “Natural History of the Hyperinsulinism Hyperammonemia Syndrome – A Multi-center Observational Study Incorporating Patient-centered Data through the HI Global Registry.” In the study, she will describe the natural history of the hyperinsulinism hyperammonemia syndrome using a composite approach that combines database and medical record reviews from US Congenital Hyperinsulinism Centers of Excellence, with telephone interviews, and HI Global Registry data.
**CHI International Pilot Grants**	
2019	CHI directly granted Amanda Ackermann, MD, PhD, of the Children’s Hospital of Philadelphia funds to study a “Novel Mouse Model to Investigate Pathophysiology of Hyperinsulinism/Hyperammonemia Syndrome.” Hyperinsulinism/hyperammonemia (HI/HA) syndrome is not only a disease of hypoglycemia. Patients with HI/HA syndrome also have high blood ammonia levels, seizures, and neurodevelopmental differences that currently are not well-understood and do not have any specific treatments. It has been difficult to study each of these features of HI/HA syndrome in patients because each one can affect the other features.

## A Lasting Infrastructure for Collaboration to Ensure Future Gains for the HI Community

To ensure a continuing cycle of innovation, and to accelerate and help advance current research projects, CHI has launched a collaborative research network (CRN) with 56 council members. Council members are researchers, clinicians, and patient leaders from 19 countries, 21 hospitals and academic institutions, five biotech companies, and three nonprofits that support people with CHI. Retired clinicians and researchers who paved the way for today’s progress and patients and caregiver leaders round out the team.

The CHI CRN is led by a core team consisting of a lead researcher and clinician and three CHI staff members. Council members have joined one of seven workstreams: *Care Guidelines/Centers of Excellence, Clinical Trials/Industry Engagement, Diagnostics, Genetics, Glucose Monitoring, Medical and Surgical Treatments, and What is HI: Nomenclature and Inclusion* (**Table 3**). Together, CRN council members are developing a patient-focused prioritized research agenda that will potentially lead to faster and more accurate diagnosis, drive new evidence-based treatments and cures, standardize clinical guidelines, and facilitate improved access to treatment, medication, and supplies (19).

**Table 3:** CHI CRN Workstreams with Mission Statements.

Workstream	Mission Statement
Care Guidelines/Centers of Excellence	To create a prioritized research agenda on the topic of care guidelines/Centers of Excellence, we envision a better future for those with CHI through improved care guidelines, centers of excellence, and collaboration for better quality of life and outcomes for patients.
Clinical Trials/Industry Engagement	To create a space where patient and industry leaders and academic researchers and clinicians can come together to consider collaborations and approaches to enable progress in clinical research for today’s projects and tomorrow’s innovations.
Diagnostics	To create a prioritized research agenda for the topic of diagnostics, we envision a better future for those with CHI through improved diagnostics for better quality of life and outcomes for patients.
Genetics	To create a prioritized research agenda for the topic of genetics, we envision a better future for those with CHI through improved understanding of genetics for better quality of life and outcomes for patients.
Glucose Monitoring	To create a prioritized research agenda for the topic of glucose monitoring, we envision a better future for those with CHI through improved glucose monitoring for better quality of life, diagnostics, and outcomes for patients.
Medical and Surgical Treatments	To create a prioritized research agenda for a better future for those with CHI through new and better medical and surgical treatments or cures.
What is HI: Nomenclature and Inclusion	To develop a plan to bring synergy to the way the patients, physicians, and medical industry decision makers describe the disease, to better define who is counted in the “CHI patient community,” to agree upon a set of terms that define the condition and its subtypes, and to educate all appropriate stakeholders.

The CHI CRN is made possible by the Chan Zuckerberg Initiative (CZI) Rare As One Network (RAO). In 2019, CHI applied and was accepted to be one of thirty CZI RAO patient advocacy organizations in the US (27). The RAO Network, which has now expanded to 50 patient organizations, is providing CHI with the knowledge to continue to develop and sustain our patient-led research initiative. Through RAO, CHI studied the work of the Castleman Disease Research Network (CDRN). The CDRN identified the limitations of a traditional “Request for Proposal (RFA)” approach to selecting research to be funded (28). The RFA method relies on a small group of people raising funds and selecting topics for funding. Often, a limited group of researchers in the field respond to the request, and the topic is determined by chance. In contrast, with the CRN approach, experts from a variety of institutions work collaboratively and deliberately, in a calculated manner to determine what research is necessary and to set priorities as one research community, focused on the needs of the patients and their families. CHI has adopted this approach and is refining it as needed for the HI Community.

**Figure 6:**
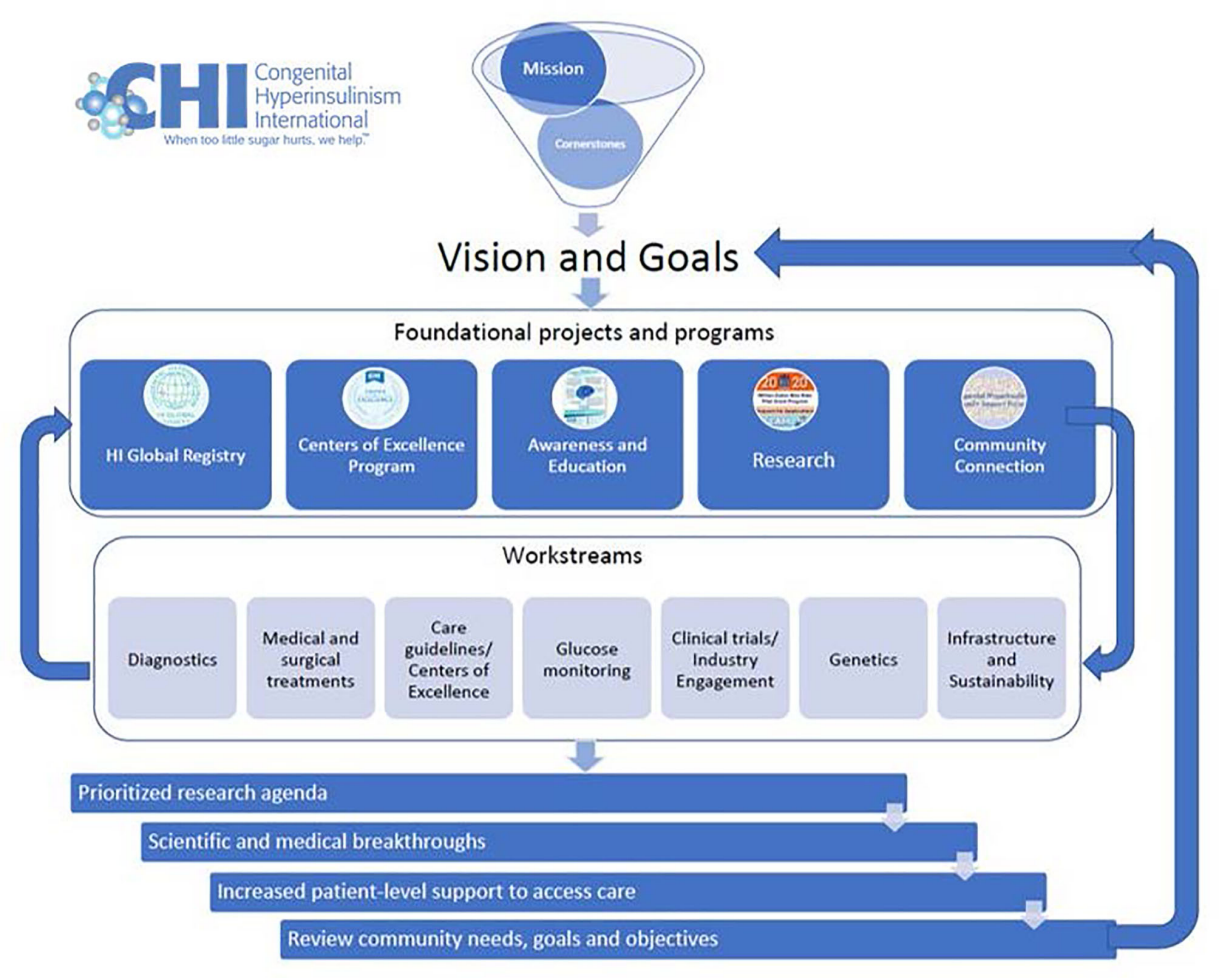
CHI Collaborative Research Network Model.

With the initial prioritized research agenda to be completed in 2022, CHI will work with its partners to create a lasting infrastructure for collaboration, with platforms for data sharing, and consistent small and large group meetings, to actualize the research projects. CHI will use the prioritized research agenda as compass and guide for its programmatic work which will continually evolve with the progress made because of CRN research breakthroughs (**Figure 6**).

## Conclusion

People with HI and their families need ongoing support and new and better treatments. CHI, with its many partners and supporters, collaborates with the world’s leading HI experts, medical professionals, technologists, and people who live with HI and their families worldwide, to make the present as livable as possible. With financial support from individual donors, foundations, and companies CHI offers extensive research, support, and awareness programs. Together with its partners CHI is creating a brighter future for those with HI, with more timely diagnosis, more access to existing treatments, the development of new and better tools and treatments, even cures, and more support to people with HI and their families. As the CHI slogan states, “When too little sugar hurts, we help.”

## Data Availability Statement

The raw data supporting the conclusions of this article will be made available by the authors, without undue reservation.

## Ethics Statement

The studies involving human participants were reviewed and approved by North Star Review Board. The patients/participants provided their written informed consent to participate in this study.

## Author Contributions

JR, SB, DT, and JS wrote the initial draft of the article. TP provided the data analysis for the HIGR section and TP and JS provided comments, critical revisions, and support with formatting. JS provided the artwork and charts. All authors contributed to the article and approved the submitted version.

## Funding

CHI receives funding from the Chan Zuckerberg Initiative (grant number: 2019-211572), The Bydale Foundation, Global Genes, Crinetics Pharmaceuticals, Eiger Biopharmaceuticals, Hanmi Pharmaceuticals, Rezolute, and Zealand Pharma, and other foundations, companies, and individual donors.

## Conflict of Interest

Authors are employees of Congenital Hyperinsulinism International (CHI) or members or former members of the CHI Board of Directors. CHI receives sponsorship for events from Amidebio, Betabionics, Crinetics Pharmaceuticals, Eiger, Hanmi, Rezolute, Twist and Zealand Pharma over the past 3 years. Crinetics Pharmaceuticals, Eiger, Hanmi Pharmaceuticals, Rezolute and Zealand Pharma are current sponsors of the HI Global Registry. None of the authors receive any fees directly from the companies. CHI staff and the board of directors retain all control of the programmatic aspects of these programs.

## Publisher’s Note

All claims expressed in this article are solely those of the authors and do not necessarily represent those of their affiliated organizations, or those of the publisher, the editors and the reviewers. Any product that may be evaluated in this article, or claim that may be made by its manufacturer, is not guaranteed or endorsed by the publisher.
